# Identifying the optimal criteria of radiotherapeutic parameters for patients with unresectable locally advanced hepatocellular carcinoma

**DOI:** 10.18632/oncotarget.5713

**Published:** 2015-10-16

**Authors:** Seok Hyun Son, Hong Seok Jang, Soo Yoon Sung, Hye Jin Kang, Sojung Lee, Chul Seung Kay

**Affiliations:** ^1^ Department of Radiation Oncology, Incheon St. Mary's Hospital, College of Medicine, The Catholic University of Korea, Seoul, Korea; ^2^ Department of Radiation Oncology, Seoul St. Mary's Hospital, College of Medicine, The Catholic University of Korea, Seoul, Korea

**Keywords:** hepatocellular carcinoma, radiotherapy, optimal criteria, radiotherapeutic parameters

## Abstract

The purpose of this study is to identify the optimal criteria of the radiotherapeutic parameters in patients with unresectable locally advanced hepatocellular carcinoma (HCC). 103 patients were enrolled in this study. All patients received RT delivered using the TomoTherapy Hi-Art system between March 2006 and February 2012. We evaluated the planning target volume (PTV), total dose (Gy_10_), and NTNL-V_BED20_ (non-target normal liver volume receiving more than a biologically effective dose of 20 Gy_8_) as significant radiotherapeutic parameters associated with hepatic function deterioration and local progression-free survival (PFS). A PTV of 279 cm^3^ or 304 cm^3^, a total dose of 60 Gy_10_, and a NTNL-V_BED20_ of 40.8% were identified as the optimal cut-off values of radiotherapeutic parameters to prevent hepatic function deterioration and prolong local PFS. Based on these findings, patients were divided in a favorable and an unfavorable prognosis group. The differences in median local PFS, overall survival, and incidence of deteriorated hepatic function between the two groups were 11.2 months, 11.1 months, and 71.7%, respectively (*p* < 0.001 in each case). In conclusion, we suggest that the optimal criteria of the radiotherapeutic parameters for patients with unresectable locally advanced HCC are: PTV ≤ 279 cm^3^, total dose > 60 Gy_10_, and NTNL-V_BED20_ ≤ 40.8%.

## INTRODUCTION

The standard treatments for unresectable hepatocellular carcinoma (HCC) are transarterial chemoembolization (TACE) and sorafenib. TACE is currently recommended for large multinodular HCC, while sorafenib is the suggested first-line of treatment for HCC with vascular invasion or extrahepatic spread. There is now strong evidence that TACE enhances the survival of patients with unresectable locally advanced HCC [1, 2]. However, large tumors have an arterial and portal blood supply, and hence, they might remain viable after TACE and give rise to recurrence or metastasis [3]. Radiotherapy (RT) in addition to TACE could overcome these limitations and improve clinical outcomes [4–9]. Although the Sorafenib Hepatocellular Carcinoma Assessment Randomized Protocol and the Asia-Pacific trial found that the sorafenib improved overall survival compared with placebo, the survival benefit was modest [10, 11]. In addition, many studies have suggested RT as an effective treatment option for patients with unresectable locally advanced HCC [12, 13]. A new strategy including RT is therefore needed in the treatment of unresectable locally advanced HCC.

However, to date, there are no clear guidelines as to when and how it should best be used. In this study, we have evaluated the clinical outcomes of RT in patients with unresectable locally advanced HCC and have identified the optimal criteria of the radiotherapeutic parameters for its use.

## RESULTS

### Response, survival and hepatic function deterioration

The median follow-up duration was 11.6 months (range: 3.5–85.3 months), and 13 patients (12.6%) were alive at the time of analysis. Complete or partial response were achieved in 58 of 103 patients (56.3%), while stable or progressive disease were observed in 45 of 103 patients (43.7%). The median local progression-free survival (PFS) duration was 9.0 months, and the 1-year and 2-year local PFS rates were 41.5% and 16.5%, respectively. The median overall PFS duration was 6.4 months, and the 1-year and 2-year overall PFS rates were 26.9% and 10.8%, respectively. The median overall survival (OS) duration was 11.6 months, and the 1-year and 2-year OS rates were 48.5% and 23.4%, respectively. Hepatic function deterioration occurred in 47 patients (45.6%).

### Identifying cut-off values of radiotherapeutic parameters associated with hepatic function deterioration and local PFS

**Figure 1:**
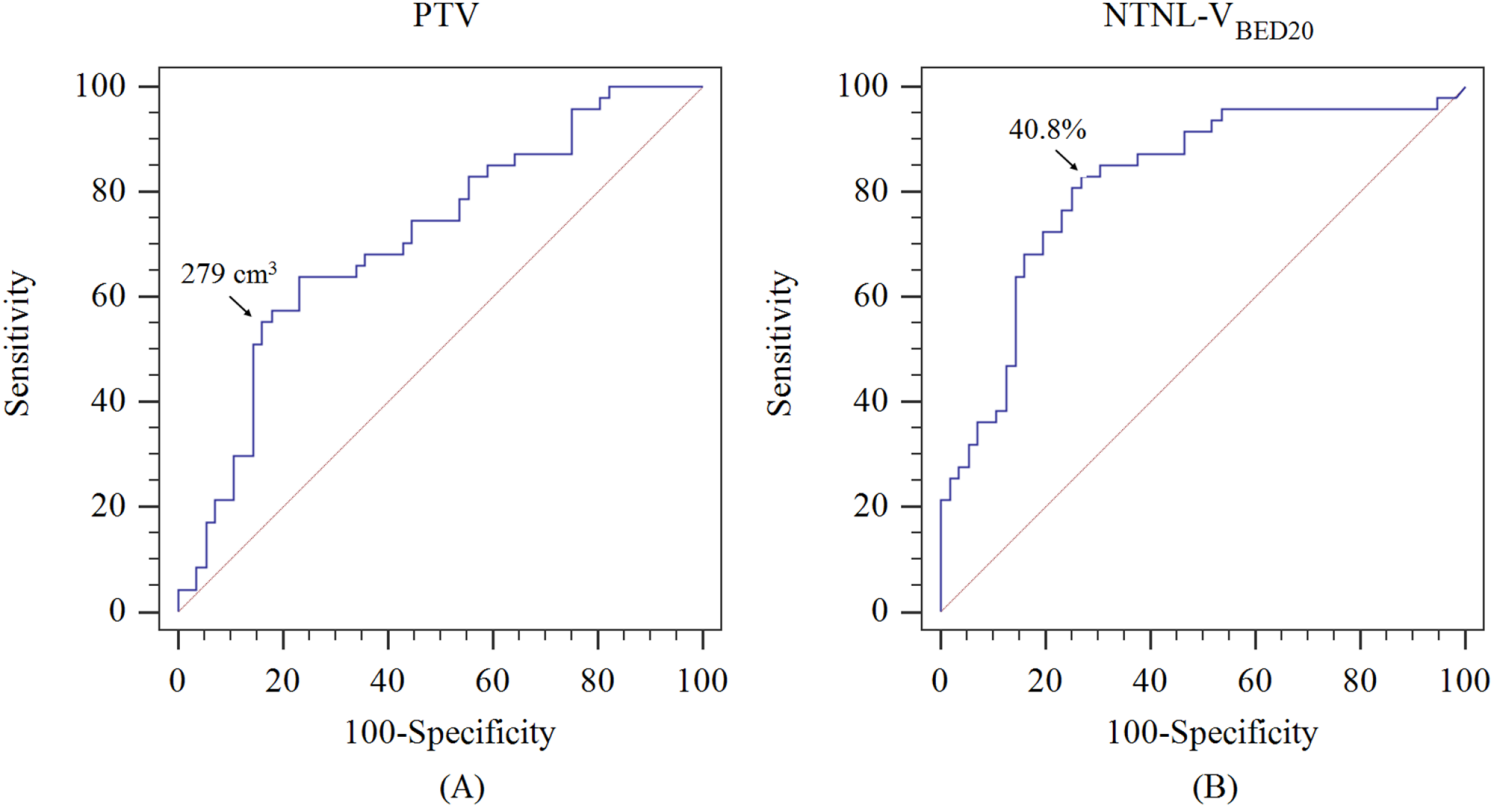
The receiver operating characteristic curves **A.** PTV associated with hepatic function deterioration, and **B.** NTNL-V_BED20_ associated with hepatic function deterioration

Based on a maximally selected chi-square test, a planning target volume (PTV) of 279 cm^3^ and a non-target normal liver (NTNL)-V_BED20_ of 40.8% were found to be significantly associated with hepatic function deterioration. These cut-off values were re-evaluated by using a receiver operating characteristics (ROC) curve. For PTV, the sensitivity was 0.553 and the specificity was 0.839, with an area under the curve (AUC) of 0.712 (*p* < 0.001). For NTNL-V_BED20_, the sensitivity was 0.830 and the specificity was 0.732, with an AUC of 0.817 (*p* < 0.001) (Figure 1). The total dose (Gy_10_) was not significantly associated with hepatic function deterioration (*p* = 0.056).

**Figure 2:**
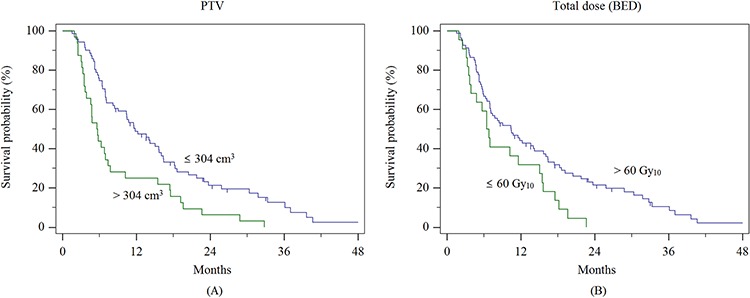
Local progression-free survival curve **A.** according to PTV, and **B.** according to total dose (BED)

Based on a maximally selected log-rank test, a PTV of 304 cm^3^ and a total dose of 60 Gy_10_ were also found to be significantly associated with local PFS, and these cut-off values were re-evaluated by using the Cox regression model (PTV of 304 cm^3^: HR = 2.092, CI = 1.353–3.234, *p* = 0.002; total dose of 60 Gy_10_: HR = 1.824, CI = 1.116–2.979, *p* = 0.017) and Kaplan-Meier survival analysis (*p* < 0.001 and 0.015, respectively) (Figure 2).

### Identifying the optimal criteria of radiotherapeutic parameters

Patients were divided in four groups (group 1, 2, 3, and 4) based on a PTV of 279 cm^3^ and a NTNL-V_BED20_ of 40.8%, which were identified as cut-off values of significant radiotherapeutic parameters associated with hepatic function deterioration. Table 1(A) shows local PFS, overall PFS, OS, and the incidence of hepatic function deterioration of these four groups. The best clinical outcomes were achieved by group 1 (with PTV ≤ 279 cm^3^ and NTNL-V_BED20_ ≤ 40.8%), while the worst outcomes were observed in group 4 (with PTV > 279 cm^3^ and NTNL-V_BED20_ > 40.8%). The differences in median local PFS, overall PFS, OS, and incidence of hepatic function deterioration between group 1 and group 4 were 10.1 months, 3.2 months, 11.3 months, and 79.2%, respectively (*p* = 0.007, 0.115, 0.057, and <0.001, respectively).

**Table 1:** Clinical outcomes in four groups associated with hepatic function deterioration and local progression-free survival

(A) Comparison of four groups associated with hepatic function deterioration					
Variables	Group 1	Group 2	Group 3	Group 4	*p* value
PTV (cm^3^)	≤279	≤279	>279	>279	
NTNL-V_BED20_ (%)	≤40.8	>40.8	≤40.8	>40.8	
Outcome					
No of patients	38	31	12	22	
Local PFS (mo)	15.7	6.9	6.3	5.6	0.007
Overall PFS (mo)	7.9	6.4	4.5	4.7	0.115
OS (mo)	15.8	16.1	8.3	4.5	0.057
Deterioration of hepatic function (%)	2.6	52.6	66.7	81.8	<0.001
**(B) Comparison of four groups associated with local progression-free survival**					
Variables	Group A	Group B	Group C	Group D	*p* value
PTV (cm^3^)	≤304	≤304	>304	>304	
Total dose (Gy_10_)	>60	≤60	>60	≤60	
Outcome					
No of patients	65	6	14	18	
Local PFS (mo)	11.8	11.6	5.7	4.8	0.005
Overall PFS (mo)	7.7	5.6	4.8	4.6	0.055
OS (mo)	16.0	11.6	7.4	6.8	0.009
Deterioration of hepatic function (%)	30.7	50.0	71.4	77.8	<0.001

*Abbreviations*: PTV = planning target volume; NTNL = non-target normal liver; BED = biologically effective dose; No = number; PFS = progression-free survival; mo = months; OS = overall survival

In addition, patients were divided in four groups (group A, B, C, and D) based on a PTV of 304 cm^3^ and a total dose of 60 Gy_10_, which were identified as cut-off values of significant radiotherapeutic parameters associated with local PFS. Table 1(B) shows local PFS, overall PFS, OS, and incidence of hepatic function deterioration in these four groups. The best clinical outcomes were achieved by patients in group A (with PTV ≤ 304 cm^3^ and total dose > 60 Gy_10_) and the worst outcomes were observed in group D (with PTV > 304 cm^3^ and total dose ≤ 60 Gy_10_). The differences in median local PFS, overall PFS, OS, and incidence of hepatic function deterioration between group A and group D were 7.0 months, 3.1 months, 9.2 months, and 47.1%, respectively (*p* = 0.005, 0.055, 0.009, and <0.001, respectively).

**Figure 3:**
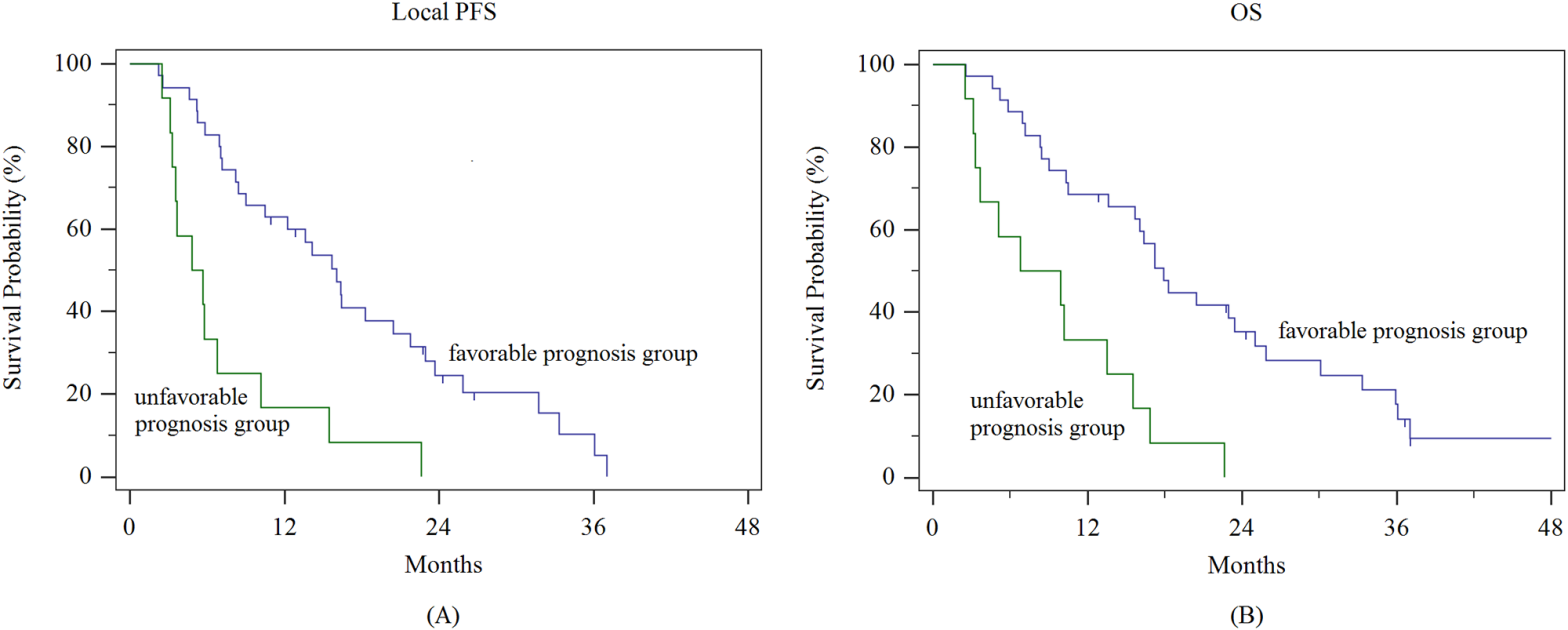
Local progression-free survival and overall survival of the favorable and unfavorable prognosis groups **A.** local progression-free survival, and **B.** overall survival

Based on these results, we merged group 1 and group A in a favorable prognosis group (with PTV ≤ 279 cm^3^, total dose > 60 Gy_10_ and NTNL-V_BED20_ ≤ 40.8%), while group 4 and group D formed an unfavorable group (with 40%) as an unfavorable group. These groups were compared with respect to local PFS, overall PFS, OS, and incidence of hepatic function deterioration (Table 2, Figure 3). The differences in median local PFS, overall PFS, OS, and incidence of hepatic function deterioration between the favorable prognosis group and unfavorable prognosis group were 11.2 months, 3.6 months, 11.1 months, and 71.7%, respectively (*p* < 0.001, 0.0215, <0.001, and <0.001, respectively).

**Table 2:** Comparison between the favorable and unfavorable prognosis groups

Variables	Favorable prognosis group	Unfavorable prognosis group	*p* value
PTV (cm^3^)	≤279	>304	
Total dose (Gy_10_)	>60	≤60	
NTNL-V_BED20_ (%)	≤40.8	>40.8	
Outcome			
No of patients	30	12	
Local PFS (mo)	16.0	4.8	<0.001
Overall PFS (mo)	7.9	4.3	0.022
OS (mo)	17.9	6.8	<0.001
Deterioration of hepatic function (%)	3.3	75	<0.001

*Abbreviations*: PTV = planning target volume; NTNL = non-target normal liver; BED = biologically effective dose; No = number; PFS = progression-free survival; mo = months; OS = overall survival

## DISCUSSION

Although the therapeutic role of RT in unresectable locally advanced HCC has not been well established, many studies have reported that it is safe and effective for the treatment of HCC patients [5–9, 13–17]. Because the improved gain of survival cannot be achieved in all patients received RT, it is important to establish the optimal criteria of radiotherapeutic parameters in order to improve its efficacy. Therefore, we attempted to identify the optimal cut-off values of radiotherapeutic parameters associated with local PFS and hepatic function deterioration to enhance its safety and efficacy. Among the possible prognostic factors, we excluded the pretreatment Child-Pugh (CP) score because only patients with a pretreatment CP score of 5–7 were considered to be eligible for RT. A PTV of 304 cm^3^ and a total dose of 60 Gy_10_ were found to be cut-off values of significant radiotheraprutic parameters associated with local PFS, and a PTV of 279 cm^3^ and a NTNL-V_BED20_ of 40.8% were found to be cut-off values of significant radiotheraprutic parameters associated with hepatic function deterioration. Based on the values of these three parameters, a favorable and an unfavorable prognosis group were defined and compared. Local PFS, overall PFS, OS, and incidence of hepatic function deterioration were all better in the favorable prognosis group than that in the unfavorable prognosis group (local PFS: 16.0 vs. 4.8 months; overall PFS: 7.9 vs. 4.3 months; OS: 17.9 vs. 6.8 months; incidence of hepatic function deterioration: 3.3% vs. 75.0%). Our results demonstrated that optimizing the selection of patients for RT resulted in an improvement in both its safety and efficacy.

Dawson *et al.* reported that the best outcomes after 3-dimensional conformal radiotherapy (3D-CRT) or stereotactic body radiotherapy (SBRT) are achieved in patients with fewer than 3 lesions that are less than 6 cm in size with intact liver function [18]. Similary, Toramatsu *et al*. found that when the nominal diameter of gross tumor volume (GTV) is more than 6.3 cm, the average risk of radiation-induced liver disease is 94.5% for intensity-modulated radiotherapy (IMRT) [19]. As for SBRT, the optimal tumor size for RT is smaller. Takeda *et al*. recommended combination therapy of TACE plus SBRT for solitary tumors with a tumor volume less than 100 cc [20]. Huang *et al*. found that OS is significantly lower in patients with tumors larger than 4 cm (HR: 0.5, *p* = 0.028) [21]. The PTV of 279 cm^3^ and 304 cm^3^ found in this study are comparable with the GTV of about 6.5–7 cm diameter, which is larger than the 4–5.5 cm diameter of tumor that is recommended for SBRT, and with the GTV of 6.3 cm recommended by Toramatsu *et al*. [19]. Chen *et al*. reported that a higher dose (>50 Gy in 2 Gy fractions) resulted in better survival (median OS, 10.5 vs. 6.9 months, *p* < 0.001) [22]. A biologically effective dose (BED) of 60 Gy_10_, which was also identified as a significant parameter in this study, corresponds to 50 Gy in 2 Gy fractions. A dose of 45–50 Gy or higher in conventional fractionations has generally been used in the 3D-CRT studies, while 30–60 Gy in 3–5 fractions, similar to or higher than a BED of 60 Gy_10_, has been used in the SBRT studies. Consistently, a BED of 60 Gy_10_ in this study was considered the minimum dose required to achieve a better local PFS. The NTNL-V_BED20_ was a significant parameter associated with hepatic function deterioration, which was identified in our previous study [23]. Liang *et al*. suggested that V_20_ is an important parameter in patients treated with hypofractionated RT (4–6 Gy per fraction) [24], and Son *et al*. demonstrated that V_15_ is a significant parameter associated with increased CP score [25]. When compared with V_20_ of 48.5% reported by Liang *et al*. and V_15_ of 43.2% reported by Son *et al*., the V_BED20_ of 40.8% found in this study could be considered adequate. Our toxicity results support the validity of this cut-off value as there was a large difference in the incidence of hepatic function deterioration between the favorable and unfavorable prognosis group (3.3% vs. 75.0%).

In addition, the values of these parameters are not fixed but can be varied in the treatment planning phase. The parameters in this study were derived from tomotherapy. When other treatment planning techniques, such as 3D-CRT and fixed-beam IMRT, are used, the distribution of low-to-moderate dose could differ from that when tomotherapy is used; moreover, the distribution of low-to-moderate dose in other treatments could affect the value of these parameters. Therefore, PTV, total dose, and NTNL-V_BED20_ all depend on the treatment techniques. The use of compression devices, gated therapy, or image-guided radiotherapy (IGRT) for reducing respiratory-induced tumor motion or allowing a more precise delivery of radiation could allow a smaller PTV to be used. The radiation dose and NTNL-V_BED20_ could also be improved by using the IG-IMRT technique instead of 3D-CRT. The values of these three parameters could be adjusted within the favorable ranges during the treatment planning.

In conclusion, we suggest that the optimal criteria of radiotherapeutic parameters for patients with unresectable locally advanced HCC are: PTV ≤ 279 cm^3^, total dose > 60 Gy_10_ and NTNL-V_BED20_ ≤ 40.8%. Because patients who meet these criteria would derive the most benefit from RT, the addition of RT to current standard modalities should be considered. Further confirmation of these findings should be sought in larger-scale studies.

## MATERIALS AND METHODS

### Patients

The inclusion criteria were: primary unresectable locally advanced HCC without distant metastasis, RT with a curative aim, age >18 years, CP score of 5, 6, or 7 within 1 month prior to RT, Eastern Cooperative Oncology Group (ECOG) performance status of 0 or 1.

A total of 103 patients were eligible for this study, all of whom received RT using the TomoTherapy Hi-Art system (TomoTherapy Inc., Madison, WI, USA), at Incheon St. Mary's Hospital and Seoul St. Mary's Hospital, between March 2006 and February 2012. Patient data were retrospectively reviewed following institutional review board approval.

Age, sex, ECOG performance status, TNM stage, pretreatment CP score, absence or presence of hepatitis or liver cirrhosis, and level of alpha-fetoprotein (AFP) were evaluated. Prior to RT, TACE was performed in 95 patients (median, 2 times; range, 1–11 times), percutaneous ethanol injection (PEI) in 8 patients (median, 2 times; range, 1–3 times), radiofrequency ablation (RFA) in 8 patients (median, 2 times; range, 1–3 times), and systemic chemotherapy in 14 patients. The patient characteristics are shown in Table 3.

**Table 3:** Clinical characteristics

Variables	*n*	(%)
Gender		
Male	80	77.7
Female	23	22.3
Age (year)		
median	59	
range	21–80	
ECOG		
0	38	36.9
1	65	63.1
Hepatitis		
None	2	1.9
HBV	73	70.9
HCV	9	8.7
Others	19	18.4
Liver cirrhosis		
No	32	31.1
Yes	71	68.9
AFP (IU/mL)		
≤400	67	65.0
>400	36	35.0
Child-Pugh score before RT		
A5	61	59.2
A6	30	29.1
B7	12	11.7
TNM stage		
II	14	13.6
III	81	78.6
IVA	8	7.8
Previous treatment		
None	7	6.8
TACE	95	92.2
RFA	8	7.8
PEI	8	7.8
Chemotherapy	14	13.6

Abbreviations: ECOG PS = Eastern Cooperative Oncology Group performance status; HBV = hepatitis B virus; HCV = hepatitis C virus; PVTT = portal vein tumor thrombosis; AFP = alpha-fetoprotein; TACE = transcarterial chemoembolization; RFA = radiofrequency ablation; PEI = percutaneous ethanol injection

### Radiotherapy

For simulation, patients were immobilized using the BodyFix system (Medical Intelligence GmbH, Schwabmunchen, Germany), in which the abdomen was compressed at low pressure using foil. A spiral computed tomography (CT) scan was then performed with intravenous contrast and a 2.5 mm slice thickness, using either a SOMATOM (Siemens, Berlin, Germany) or a LightSpeed RT16 (GE, Waukesha, WI, USA) CT scanner.

The GTV was defined as the tumor volume enhanced in the arterial phase and diluted in the delayed phase of the CT scan. The PTV was generated by adding 5–15 mm to the GTV in 71 of the 103 patients, facilitating asymmetric margin expansion in order to reduce irradiation to the stomach, duodenum, and small intestine. In the remaining 32 patients, 4-dimensional (4D) CT was performed to generate the internal target volume in order to compensate for respiration-induced liver movement due to the installation of 4D-CT in March 2009 at Seoul St. Mary's Hospital and in March 2011 at Incheon St. Mary's Hospital. Organs at risk, such as the total liver, NTNL, stomach, duodenum, intestine, kidney, and spinal cord, were also contoured for evaluation of the irradiated dose. The NTNL volume was defined as the total liver volume minus the PTV.

The PTV was 330.1 ± 275.1 cm^3^, and the normal liver volume was 1209.7 ± 426.9 cm^3^. The dose per fraction to the PTV was 1.8–5 Gy, and the total dose was 40–60 Gy (median: 50 Gy), prescribed to 95% of the PTV. Forty-one patients (39.8%) were treated with 1.8–2.5 Gy per fraction, while 62 patients (60.2%) were treated with 4–5 Gy per fraction. The total dose delivered to the target was converted to a BED, based on the linear-quadratic model with an α/β ratio of 10, and the total dose was 50.5–82.5 Gy_10_ (median: 73.5 Gy_10_). The α/β ratio of the normal liver is unclear. In our previous study, we determined the α/β ratio of the normal liver by comparing the incidence of hepatic function deterioration between 2 different fractionation schemes of RT [23]. Therefore, an α/β ratio of 8 was used for calculating the BED of the normal liver in this study. NTNL-V_BED20_, which is the fraction of the NTNL volume receiving more than a BED of 20 Gy_8_, was identified as a predictive parameter for hepatic function deterioration [23]. NTNL-V_BED20_ was 10.6–79.3% (median: 45.4%).

Treatment planning utilized the built-in software of the TomoTherapy Planning Station, which was used for the TomoTherapy Hi-Art system. We evaluated the dose-volume histogram and the dose distributions slice by slice. We then approved the treatment plan if the tumor coverage was adequate and the doses to the surrounding normal tissues were within acceptable limits. Megavoltage cone-beam CT was performed during each treatment session before actual beam delivery. The patient set-up and position were corrected using automated image registration, and the anatomical accuracy was always evaluated by a radiation oncologist.

### Evaluation and statistical analysis

Tumor response was defined as the best response in the dynamic CT scans obtained 1 month and 3 months after RT, according to the modified Response Evaluation Criteria in Solid Tumors [27]. PFS was measured from the date of RT to the date of progression or the last follow-up. OS was measured from the date of RT to the date of death or the last follow-up.

We considered local PFS and hepatic function deterioration as clinically relevant responses to RT. Hepatic function deterioration was defined as an increase of at least 2 points in the CP score within 3 months after completing RT. An increase in the CP score reflects hepatic function deterioration, and this increase has been used for the assessment of hepatic toxicity after treatment for liver diseases [23, 25–28].

We evaluated the PTV, NTNL-V_BED20_, and total dose (Gy_10_) as significant parameters affecting the hepatic function. Maximally selected chi-square test was used for evaluating PTV, NTNL-V_BED20_, and total dose (Gy_10_) and identifing their cut-off values. The results were re-evaluated by using a ROC curve. We also evaluated PTV and total dose (Gy_10_) as significant parameters affecting local PFS. The cut-off values were calculated using maximally selected log-rank test and the results were re-evaluated with the Cox regression model and Kaplan-Meier survival analysis.

Based on these results, patients were divided in four groups according to the cut-off values of radiotherapeutic parameters associated with local PFS or hepatic function deterioration, respectively, and local PFS, overall PFS, OS and incidence of hepatic function deterioration were compared. Then, by combination of these groups, patients were finally divided in two groups, with either favorable or unfavorable prognosis group, and these two groups were then compared in terms of local PFS, overall PFS, OS, and incidence of hepatic function deterioration.

Statistical analysis was performed using R version 3.1.2 (R Development Core Team, Vienna, Austria) and MedCalc version 14.12 (MedCalc Software bvba, Ostend, Belgium), and *p* values <0.05 were considered to be statistically significant.
